# The streptococcal collagen-like protein-1 (Scl1) is a significant determinant for biofilm formation by group a *Streptococcus*

**DOI:** 10.1186/1471-2180-11-262

**Published:** 2011-12-14

**Authors:** Heaven A Oliver-Kozup, Meenal Elliott, Beth A Bachert, Karen H Martin, Sean D Reid, Diane E Schwegler-Berry, Brett J Green, Slawomir Lukomski

**Affiliations:** 1 Department of Microbiology, Immunology, and Cell Biology, West Virginia University School of Medicine, Morgantown, WV 26506, USA; 2 Mary Babb Randolph Cancer Center, West Virginia University School of Medicine, Morgantown, WV 26506, USA; 3 Microscope Imaging Facility, West Virginia University School of Medicine, Morgantown, WV 26506, USA; 4 Department of Microbiology and Immunology, Wake Forest University School of Medicine, Winston-Salem, NC 27157, USA; 5 Pathology and Physiology Research Branch, National Institute of Occupational Safety and Health (NIOSH), Morgantown, WV 26505, USA; 6 Allergy and Clinical Immunology Branch, Health Effects Laboratory Division, National Institute of Occupational Safety and Health (NIOSH), Morgantown, WV 26505, USA; 7 Beth A. Bachert was enrolled in Biomedical Sciences Graduate Programs, West Virginia University Health Sciences Center

## Abstract

**Background:**

Group A *Streptococcus *(GAS) is a human-specific pathogen responsible for a number of diseases characterized by a wide range of clinical manifestations. During host colonization GAS-cell aggregates or microcolonies are observed in tissues. GAS biofilm, which is an *in vitro *equivalent of tissue microcolony, has only recently been studied and little is known about the specific surface determinants that aid biofilm formation. In this study, we demonstrate that surface-associated streptococcal collagen-like protein-1 (Scl1) plays an important role in GAS biofilm formation.

**Results:**

Biofilm formation by M1-, M3-, M28-, and M41-type GAS strains, representing an intraspecies breadth, were analyzed spectrophotometrically following crystal violet staining, and characterized using confocal and field emission scanning electron microscopy. The M41-type strain formed the most robust biofilm under static conditions, followed by M28- and M1-type strains, while the M3-type strains analyzed here did not form biofilm under the same experimental conditions. Differences in architecture and cell-surface morphology were observed in biofilms formed by the M1- and M41-wild-type strains, accompanied by varying amounts of deposited extracellular matrix and differences in cell-to-cell junctions within each biofilm. Importantly, all Scl1-negative mutants examined showed significantly decreased ability to form biofilm *in vitro*. Furthermore, the Scl1 protein expressed on the surface of a heterologous host, *Lactococcus lactis*, was sufficient to induce biofilm formation by this organism.

**Conclusions:**

Overall, this work (i) identifies variations in biofilm formation capacity among pathogenically different GAS strains, (ii) identifies GAS surface properties that may aid in biofilm stability and, (iii) establishes that the Scl1 surface protein is an important determinant of GAS biofilm, which is sufficient to enable biofilm formation in the heterologous host *Lactococcus*. In summary, the GAS surface adhesin Scl1 may have an important role in biofilm-associated pathogenicity.

## Background

Microbial biofilm formation is an important virulence mechanism, which allows immune evasion and survival against antibiotic treatments [1,2]. Many bacterial nosocomial infections are associated with biofilms formed on contaminated medical devices. Dispersal of biofilm has also been proposed to augment infection spread [3-8]. For group A *Streptococcus *(GAS), biofilm research is an emerging field and little is known about the specific surface determinants that aid in biofilm formation. GAS is characteristically associated with significant human morbidity and it is responsible for the clinically common superficial throat and skin infections, such as pharyngitis and impetigo, as well as invasive soft tissue and blood infections like necrotizing fasciitis and toxic shock syndrome [9]. Although GAS biofilm has not been associated with implanted medical devices, tissue microcolonies of GAS encased in an extracellular matrix were demonstrated in human clinical specimens [10]. Studies reported to date support the involvement of GAS surface components in biofilm formation, including the M and M-like proteins, hyaluronic acid capsule, pili and lipoteichoic acid [11-13]. As shown by Cho and Caparon [11], multiple genes are upregulated during biofilm formation and development, including the streptococcal collagen-like protein-1 (Scl1).

The *scl1 *gene encoding the Scl1 protein has been found in every GAS strain investigated and its transcription is positively regulated by Mga [14-18], indicating that Scl1 is co-expressed with a number of proven virulence factors. Structurally, the extracellular portion of Scl1 protein extends from the GAS surface as a homotrimeric molecule composed of distinct domains that include the most outward N-terminal variable (V) region and the adjacent collagen-like (CL) region composed of repeating GlyXaaYaa (GXY) sequence. The linker (L) region is close to the cell surface and contains a series of conserved direct repeats. The Scl1 protein can bind selected human extracellular matrix components [19] and cellular integrin receptors [20-22], as well as plasma components [23-27].

In this study, we investigated the importance of Scl1 in GAS biofilm using defined isogenic wild-type and *scl1*-inactivated mutant strains of GAS. We report that (i) the pathogenically diverse M41-, M28-, M3- and M1-type GAS wild-type strains have varying capacities to produce biofilm on an abiotic surface; (ii) Scl1 plays an important role during the main stages of biofilm formation with Scl1-negative mutants having an abrogated capacity for adhesion, microcolony formation and biofilm maturation; and (iii) variations in surface morphology as well as in extracellular matrix associated with bacterial cells suggest two distinct but plausible mechanisms that potentially stabilize bacterial microcolonies. We additionally show that expression of Scl1 in *Lactococcus lactis *is sufficient to support a biofilm phenotype. Overall, this work reveals a significant role for the Scl1 protein as a cell-surface component during GAS biofilm formation among pathogenically varying strains.

## Results

### Wild-type GAS strains have heterogeneous capacity for biofilm formation on abiotic surfaces

**Figure 1:**
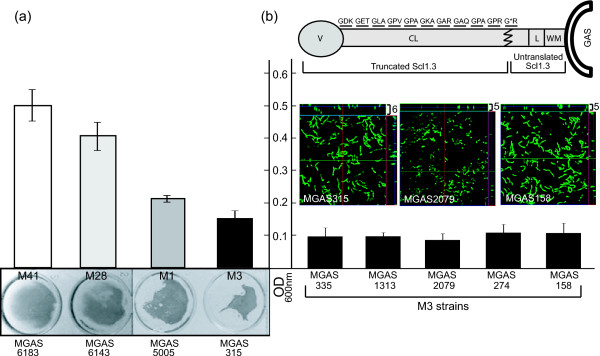
**Variation in biofilm formation among GAS strains**. (**a**) Wild type M41-, M28-, M3-, and M1-type GAS strains were grown 24 h under static conditions and analyzed spectrophotometrically following crystal violet staining (top). Visual representation of corresponding wells is shown below. (**b**) Schematic representation (not to scale) of Scl1.3 protein of M3-type GAS. Translated GXY repeats within the collagen-like (CL) region are shown with an asterisk representing the location of the premature stop codon resulting in a truncated protein. V, variable region; L, linker region; WM, wall-membrane associated region. Below, spectrophotometric measurements of 24-h biofilms following crystal violet staining are graphed for M3-type GAS strains. Absorbance values (OD_600_) are averages of at least three experiments done in triplicate wells. Corresponding confocal analyses of 24-h biofilms of MGAS315, MGAS2079, and MGAS158 are shown. Images are X-Y orthogonal Z-stack views and average vertical thickness is indicated in micrometers (top right).

Biofilm formation was compared between M41-, M28-, M3- and M1-type GAS strains representing distinct epidemiological traits (Figure 1). To assess biofilm formation after 24 h, we used spectrophotometric measurements recorded following crystal violet staining (Figure 1a). Both the M41- and M28-type strains produced more biomass as compared with M1 strain. Furthermore, the M3-type strain produced the lowest absorbance values in a crystal violet assay, indicative of lower cell biomass, as compared with the other wild-type strains. These experiments confirm previous observations [1,28] that GAS strains have varying capacity to form biofilm *in vitro*.

The failure of M3-type strain MGAS315 to produce substantial cellular biomass in the above assay was intriguing because sequence analysis of the *scl1.3 *allele found in MGAS315 revealed the presence of a TAA stop codon in the 11th GXY repeat of the Scl1.3-CL region containing a total of 25 GXY triplets [29]. This premature stop codon results in a truncated Scl1.3 variant composed of 102 amino acids (~11.4 kDa), which does not contain the cell wall-membrane (WM) associated region, thus, preventing it from anchoring to the bacterial cell surface (Figure 1b). This prompted us to investigate the biofilm formation by five additional M3-type strains, all harboring the same *scl1.3 *allele. Five additional M3-type strains, MGAS335, MGAS1313, MGAS2079, MGAS274 and MGAS158, all harboring the same *scl1.3 *allele [29] also produced poor biofilm under static conditions, as measured by crystal violet staining. Confocal laser scanning microscopy (CLSM) of three representative strains (MGAS315, MGAS2079, and MGAS158) corroborated results obtained from the crystal violet assay, indicating that these M3-type strains lack the ability to form appreciable biofilm structure. Our data suggest that the lack of capacity for biofilm-formation among M3-type GAS strains examined here might be correlated, at least in part, with lack of surface-attached Scl1.3 protein.

### Microscopic evaluation reveals differences in biofilm surface morphology

**Figure 2:**
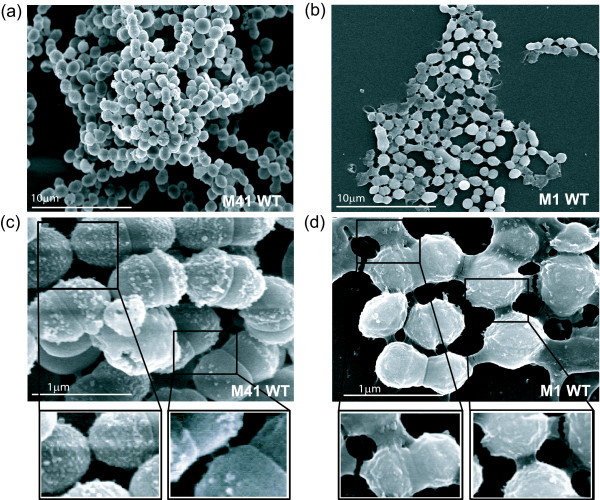
**Biofilm formation by wild type and *scl1*-inactivated isogenic mutants**. Crystal violet staining and confocal laser scanning microscopy (CLSM) of the GFP-expressing GAS were used to compare biofilm formation by GAS strains. Wild type (WT) M41-, M28-, and M1-type strains, *scl1*-inactivated mutants (*scl1*), and M41 mutant complemented for Scl1.41 expression (M41 C) were used. (**a**-**c**) Isogenic GAS strains were grown under static conditions for 24 h and bacterial biomass was detected spectrophotometrically at indicated time points following crystal violet staining. Absorbance values at OD_600 _are representative of at least three experiments performed in quadruplicate. Statistical significance is denoted as *P ≤ 0.05 and **P ≤ 0.001. (**d**-**f**) CLSM analysis of corresponding 24 h biofilms from same experiment. Images are X-Y orthogonal Z-stack views of WT (top) and mutant (bottom) GAS strains. Views are representative of ten images within a single experiment. Average vertical biofilm thickness is indicated in micrometers (top right).

**Figure 3:**
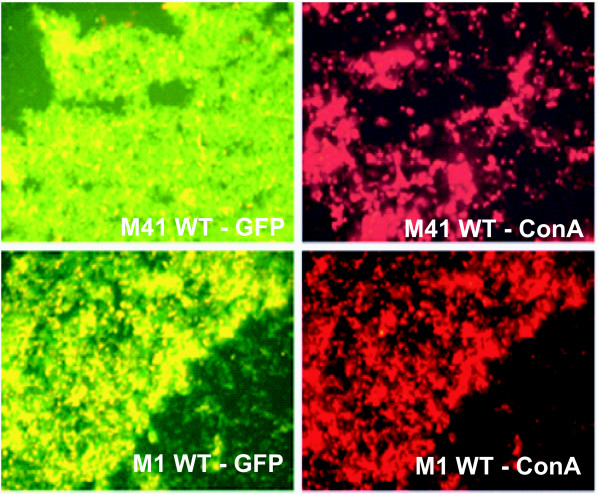
**Field emission scanning electron microscopy of GAS biofilms**. 24-h biofilms of the M1- and M41-type GAS strains were grown on glass cover slips and analyzed by FESEM. (**a**-**b**) Architecture of GAS microcolonies shown at low magnification. (**c**-**d**) Cell surface morphology and cell-to-cell junctions observed at higher magnification. Enlargements of cell-to-cell junctions are shown below.

We next conducted microscopic analysis of the biofilms formed by the wild-type (WT) M41-, M28-, and M1-type GAS strains. First, we examined the overall structural characteristics of biofilms formed after 24 h using CLSM (Figure 2d-f; Additional file 1: Figure S1a-f). The average biofilm thickness (see Methods section) differed among all three strains with M1 producing considerably thinner biofilm (mean value of 9 μm) compared to M28 (12 μm) and M41 (15 μm), a result consistent with lower spectrophotometric absorbance values (Figure 1a). In addition to measured differences in biofilm thickness, closer examination of the X-Y orthogonal Z-stack views, representing biofilm cross-sections, revealed architectural differences among the M41, M28, and M1 biofilms. The M1 biofilm, although the thinnest, seems to consist of densely-packed cells that form continuous layers, while the M28 and especially M41 biofilms seem to be less dense but exhibit more elevated supracellular assembly. We therefore used field emission scanning electron microscopy (FESEM) to define more accurately these supracellular differences observed by CLSM between the biofilms produced by the WT M1 and M41 GAS (Figure 3). FESEM exposed notable architectural differences between biofilms formed by these two strains. The M41 (Figure 3, panel a) biofilm was characterized by more diverse surface architecture with the evidence of depressions or crypts, whereas the M1 biofilm (panel b) seems to lack such pronounced surface characteristics. At higher magnification, the M41 cells have a studded cell surface morphology with protrusions linking both sister cells and cells in adjacent chains (panel c). In contrast, the M1 cells had a relatively smoother appearance likely due to the rich bacterial-associated extracellular matrix (BAEM) surrounding these cells and covering their surface (panel d). BAEM material, which was clearly seen at higher resolution between the M1-type cells, was not as evident between cells of the M41-type GAS.

### GAS biofilms differ in production of bacterial-associated extracellular matrix

**Figure 4:**
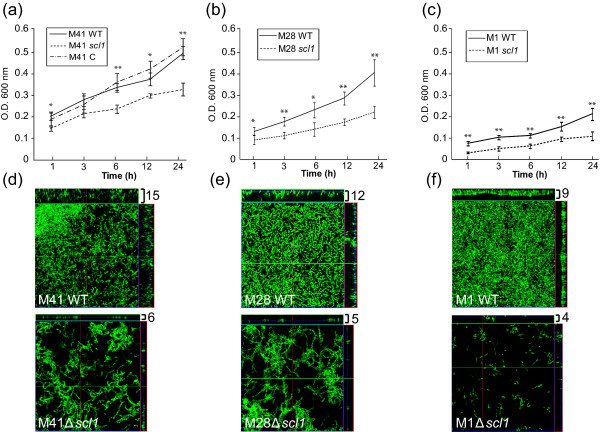
**Production of bacterial-associated extracellular matrix**. GFP-expressing wild type (WT) M41- and M1-type GAS strains were grown on glass cover slips for 24 h and stained with TRITC-conjugated concanavalin A (ConA). Confocal laser scanning microscopic (CLSM) images were separated to represent green GFP-expressing GAS cells (left images) and red ConA-TRITC staining (right images) for detection of extracellular matrix associated with each strain. Images are from one representative experiment.

The production of BAEM has been shown to be an integral component in the structural integrity of a biofilm, imparting protection from dehydration, host immune attack, and antibiotic sensitivity [30,31]. GAS cells encased in a glycocalyx were first identified by Akiyama *et al. *in skin biopsies obtained from impetigo patients. We therefore compared the production of BAEM within biofilms employing GFP-expressing GAS strains of the M1 and M41 type (Figure 4). Cells were grown to form biofilms on glass cover slips for 24 h and stained with TRITC-concanavalin A (ConA), a fluorescently-labeled lectin that binds to the extracellular polysaccharides in biofilms [32]. Fluorescent microscopy was performed to compare matrix production (red staining) by GAS strains (green). Visual screening of both biofilms indicated that the M41-type strain formed a more dispersed extracellular matrix as compared to the M1 strain, which had a dense, more closely associated matrix. In addition, averages of at least 10 fields of ConA stained matrix by CLSM support our FESEM observations that more BAEM is deposited within the biofilm by the M1 GAS cells as compared to M41 GAS. This is in agreement with the report from Akiyama *et al *that showed a substantial FITC-ConA stained matrix associated with T1-type GAS microcolonies *in vivo *and *in vitro *[10].

### Scl1 protein significantly contributes to biofilm formation by GAS

Variations in GAS pathogenicity and capacity to form biofilm are driven by specific proteins and components present on the cell surface or are secreted by the organism. It has been shown that deletion of the M and M-like surface proteins or capsule, as well as increased expression of the secreted SpeB protease decreases biofilm formation dramatically for some strains of GAS [12,33,34]. Therefore, we investigated the role of Scl1 in biofilm formation by comparing biofilms formed by GAS WT and *scl1*-inactivated (Δ*scl1*) mutant strains (Figure 2; Additional file 1: Figure S1a-f). Bacterial biomass was evaluated spectrophotometrically following crystal violet staining at 1, 6, 12, and 24 h time points, representing different stages of biofilm formation, and absorbance values rendered for the WT and Δ*scl1 *isogenic mutant strains were compared. The M41Δ*scl1 *mutant showed a 29-35% decrease in biofilm formation (the OD_600 _value obtained for the WT strain at each time point was considered 100%), which was sustained throughout all time points. This reduction was statistically significant at initial adherence (1 h), as well as during biofilm development (6-12 h) and at maturation (24 h) (Figure 2a; *P *≤ 0.05 at 1 and 12 h, *P *≤ 0.001 at 6 and 24 h). Complementation of Scl1.41 expression in the M41Δ*scl1 *mutant (M41 C) restored its ability to form biofilm to WT levels. Similarly, the M28Δ*scl1 *mutant had a significantly decreased capacity for biofilm formation in the range of 29-44% as compared to WT strain (Figure 2b; *P *≤ 0.05 at 1 and 6 h, *P *≤ 0.001 at 3, 12 and 24 h). Likewise, there was a statistically significant decrease in M1Δ*scl1 *biofilm biomass by ~42-75% compared to the WT strain (Figure 2c; *P *≤ 0.001 at 1-24 h). CLSM analysis of corresponding 24-h biofilms of these strains confirmed our crystal violet staining results at 24 h. The Δ*scl1 *mutants had substantially decreased average biofilm thickness by more than 50% (mean values) as compared to the parental WT organisms (Figure 2d-f). While these low average biofilm thickness values measured for the M1Δ*scl41 *(6 μM) and M28Δ*scl1 *(5 μM) correspond to residual biofilms made by those mutants (Additional file 1: Figure S1a-d), by comparison, the M1Δ*scl1 *(4 μM) was shown not to produce a continuous biofilm layer under these conditions (Additional file 1: Figure S1e-f). Our data support the hypothesis that the Scl1 protein plays an important functional role during GAS biofilm formation and that Scl1 contribution varies among GAS strains with different genetic backgrounds.

### Scl1 expression affects surface hydrophobicity

The surface hydrophobicity of GAS has been shown to influence the adherence to abiotic surfaces. The presence of pili [13], M and M-like proteins, and lipoteichoic acid contributes to cell surface hydrophobic properties [12,35], which in turn may influence biofilm formation by GAS. Here, we have investigated the contribution of Scl1 to surface hydrophobicity of M41-, M28-, and M1-type GAS strains using a modified hexadecane binding assay [12,36,37]. As shown in Table 1, the M28-type GAS strain MGAS6143 gave the highest actual hydrophobicity value of 94.3 ± 0.73, followed by the M41-type strain MGAS6183 (92.6 ± 0.86). In contrast, the overall surface hydrophobicity of the M1-type GAS strain MGAS5005 (80.3 ± 0.89) was significantly lower compared to both M28 and M41 strains (*P *≤ 0.001 for each comparison). Inactivation of *scl1.41 *in M41-type GAS resulted in a modest, although statistically significant, reduction in the hydrophobicity index (100% for WT vs. 92% for mutant, *P *≤ 0.001). *In-trans *complementation of the Scl1.41 expression in M41Δ*scl1*-C restored the hydrophobic phenotype of the cells to WT level (hydrophobicity index ~105%). In comparison, the contribution of the Scl1.1 and Scl1.28 proteins to surface hydrophobicity is more substantial, as evidenced by a ~21% and ~22% reduction of the hydrophobicity indices of the mutants as compared to the corresponding WT strains, respectively (*P *≤ 0.001 for both). Thus, the Scl1-mediated GAS-cell surface hydrophobicity reported here may contribute to the ability of this organism to form biofilm, as suggested for other cell surface components [12,35].

**Table 1:** Cell surface hydrophobicity of GAS strains

GAS Strain	M-Type	**Actual Value**^†^	**Hydrophobicity Index**^‡^
MGAS6183 WT	M41	92.6 ± .86	100
MGAS6183 Δ*scl1*	M41	85.2 ± 2.2	**92
MGAS6183 Δ*scl1-C*	M41	98.0 ± .31	105

MGAS5005 WT	M1	80.3 ± .89	100
MGAS5005 Δ*scl1*	M1	63.3 ± 3.2	**79

MGAS6143 WT	M28	94.3 ± .73	100
MGAS6143 Δ*scl1*	M28	72.6 ± .62	**78

^† ^Actual hydrophobicity values were calculated based on hexadecane binding as described in Methods. Values are representative of three separate experiments with ten replicates ± SD

^‡ ^Hydrophobicity Index represents the ration of actual hydrophobicity value for each strain to that of the isogenic wild-type (WT) strain multiplied by 100

** Asterisks denote a statistically significant difference of Δ*scl1 *mutants versus WTs at P ≤ 0.001

### Scl1 is sufficient to support biofilm formation in *Lactococcus lactis*

**Figure 5:**
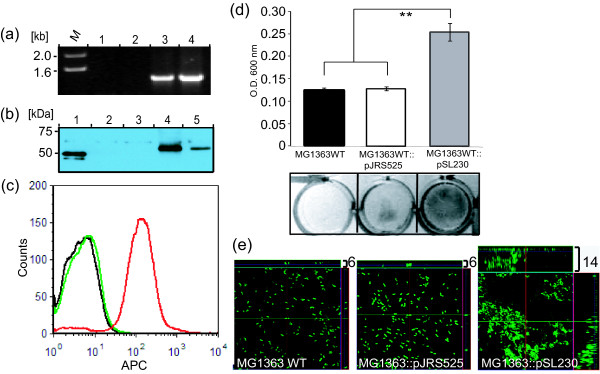
**Scl1 expression in *L. lactis *promotes biofilm formation**. *L. lactis *was transformed with the plasmid construct pSL230 to express Scl1.41 surface protein or with pJRS525 vector. (**a**) PCR analysis of *L. lactis *transformants using *scl1.41*-gene-specific primers; lanes: (1) MG1363 wild-type (WT) cells; (2) MG1363::pJRS525 vector-only control; (3) MG1363::pSL230 transformant; (4) control pSL230 plasmid DNA. (**b**) Scl1.41 expression by western blot analysis of cell-wall extracts prepared from transformed *L. lactis *and control GAS strains using anti- P176 (rScl1.41) antibodies; lanes: (1) purified recombinant P176 protein (truncated Scl1.41); (2) MG1363 WT strain; (3) MG1363::pJRS525 vector; (4) MG1363::pSL230 transformant; (5) MGAS6183 (M41) control. (**c**) Analysis of Sc1.41 expression by flow cytometry with anti-P176 (rScl1.41) rabbit polyclonal antibodies on the surface of MGAS1363 WT strain (black trace), MGAS1363::pJRS525 vector-only control (green trace) and MG1363:pSL230 transformant (red trace). (**d**) Crystal violet staining of 24 h biofilms formed by *L. lactis *WT strain, MG1363::pJRS525 vector-only control or MG1363::pSL230 transformant (top) with visual representation of the corresponding wells (bottom). Statistical significance is denoted as **P ≤ 0.001. (**e**) CLSM analysis of 24 h biofilms from same experiment shown in (**d**). Images are X-Y orthogonal Z-stack views representative of ten images within a single experiment. Average vertical biofilm thickness is indicated in micrometers (top right).

To assess whether Scl1 expression is sufficient to confer the ability for biofilm formation, we chose to express this protein in a heterologous *L. lactis *system [38,39]. The wild-type *L. lactis *strain MG1363 was transformed with plasmid pSL230 encoding the Scl1.41 protein [22] or with the shuttle vector pJRS525 alone. As shown in Figure 5a, PCR amplification of the *scl1.41 *gene employing specific primers yielded no product from the WT *L. lactis *MG1363 (lane 1) and the MG1363::pJRS525 transformant (lane 2). A product of the expected size of 1.4 kb was amplified from the pSL230 plasmid DNA control (lane 4,) as well as was amplified from the MG1363::pSL230 transformant (lane 3). Surface expression of Scl1.41 was confirmed by immunoblot analysis of cell-wall extracts prepared from *L. lactis *WT, and the MG1363::pJRS525 and MG1363::pSL230 transformants, as well as MGAS6163 (WT M41 GAS). As shown in Figure 5b, rabbit antiserum raised against purified recombinant Scl1.41 protein P176 lacking the WM region detected the corresponding immunogen (lane 1), and the homologous full length protein in cell-wall extracts of MGAS6183 (lane 5) as well as MG1363::pSL230 *L. lactis *transformant (lane 4). This band was absent in cell-wall extracts prepared from the WT *L. lactis *MG1363 (lane 2) and MG1363::pJRS525 transformant (lane 3). Expression of Scl1.41 at the cell surface was further established by flow cytometry. Rabbit anti-p176 antibodies stained Scl1.41 MG1363::pSL230 transformant, confirming the expression of Scl1.41 protein at the cell surface in the heterologous host *L. lactis *(Figure 5c, red trace). This protein was absent at the surface of WT MG1363 (black trace) and MG1363::pJRS525 transformant (green trace).

The capacity of *L. lactis *expressing Scl1.41 to form biofilm was evaluated spectrophotometrically following crystal violet staining. As shown in Figure 5d, the MG1363::pSL230 transformant demonstrated a significant increase in biofilm-associated biomass at 24 h, as compared to wild type *L. lactis *or *L. lactis*-containing pJRS525 vector (*P *≤ 0.001). Crystal violet stained wells were photographed for visual representation of biofilm formation prior to spectrophotometric assay. Biofilm thickness and architecture were evaluated by CLSM (Figure 5e; Additional file 1: Figure S2a-c). The MG1363::pSL230 transformant produced a substantially thicker biofilm (14 μm) as compared to both MG1363 WT (6 μm) and the vector-only transformant MG1363::pJRS525 (6 μm). The MG1363::pSL230 cells formed highly aggregated structures, thus, acquiring a phenotype consistent with biofilm formation. As shown in Table 2, the MG1363::pSL230 transformant, expressing Scl1.41 surface protein, had significantly enhanced cell surface hydrophobicity (hydrophobicity index of ~137% vs. 100% WT, *P *≤ 0.001) with an actual value of 82.0 ± 2.6, when compared to the MG1363 WT (59.7 ± 7.2) and the vector-only MGAS1363::pJRS525 control (56.6 ± 5.5). These data suggest a direct relationship between Scl1 expression and cell surface hydrophobicity and establish their involvement in the microorganism's ability to form biofilm *in vitro*.

**Table 2:** Cell surface hydrophobicity of *Lactococcus *strains

*Lactococcus *Strain	**Actual Value**^†^	**Hydrophobicity Index**^‡^
*L. lactis *1363 WT	59.7 ± 7.2	100
*L. lactis *1363::pJRS525	56.6 ± 5.5	98
*L. lactis *1363::pSl230	82.0 ± 2.6	**137

^† ^Actual hydrophobicity values were calculated based on hexadecane binding as described in Methods. Values are representative of three separate experiments with ten replicates ± SD

^‡ ^Hydrophobicity Index represents the ration of actual hydrophobicity value for each strain to that of the isogenic wild-type (WT) strain multiplied by 100

** Asterisks denote a statistically significant difference of Δ*scl1 *mutants versus WTs at P ≤ 0.001

## Discussion

Group A *Streptococcus *strains vary because of the vast number of M-protein types, and this variation is associated with varying frequency of isolation and exacerbation of disease [40,41]. The M41-, M28-, M3-, and M1-type strains selected for the current study represent a significant intraspecies diversity among clinical isolates of GAS. M41 GAS was a major causative agent of superficial skin infections [42-44], and strain MGAS6183, harboring the Scl1.41 protein, has been studied extensively [19,21,22]. M28-type GAS (strain MGAS6143) has historically been associated with puerperal fever and currently is responsible for extensive human infections world-wide [45]. M1T1 GAS, represented by strain MGAS5005, is a globally disseminated clone responsible for both pharyngitis and invasive infections [46-48]. The M3-type strains of GAS cause a disproportionally large number of invasive GAS infections that are responsible for traumatic morbidity and death [49,50].

Initial studies by Lembke *et al. *that characterized biofilm formation among various M types of GAS typically included several strains of the same M type [1,28]. These studies reported a significant strain-to-strain variation in ability to form biofilms within each M type. Studies that followed compared biofilm formation by defined isogenic WT and mutant strains to assess the contribution of specific GAS surface components responsible for a biofilm phenotype, including M and M-like proteins, hyaluronic acid capsule, lipoteichoic acid, and pili [12,13]. In the current study, we have assessed the role and contribution of the surface protein Scl1 in the ability to support biofilm formation by GAS strains of four distinct M types.

Recent advances in molecular mega- and pathogenomics has enabled the characterization of numerous M3-type strains with a single nucleotide resolution [51,52]. Interestingly, all five M3-type strains MGAS158, 274, 315, 335, and 1313 that were originally used for *scl1*-gene sequencing [14], plus an additional strain MGAS2079 (not reported) harbor the same *scl1.3 *allele containing a null mutation that would result in secretion of a truncated Scl1.3-protein variant. Here, we demonstrate that these GAS strains do not form biofilm on an abiotic surface. Recently, bioinformatic screening of the sequences of ~250 invasive M3-type strains isolated globally, has led to the detection of this single nucleotide polymorphism that results in disruption of Scl1.3 protein (Steve Beres and Jim Musser, personal communication). Lembke *et al. *reported heterogeneous biofilm formation among four M3-type GAS strains examined over a 24, 48, and 72-h period [28]. Biofilm was detected for one strain at a 48 h time point, on a fibrinogen-coated surface; however, it is not known whether this clinical isolate forms biofilm on abiotic surface, whether it expresses the truncated or full-length Scl1.3 protein, and whether it produces an unknown fibrinogen-binding protein, which could augment the attachment and biofilm formation. Therefore, additional studies are necessary to define the contributions of other biofilm-formation determinants in M3-type strains.

Inasmuch as, variation in biofilm formation among GAS isolates of the same M-type has been established, the molecular basis of this phenotypic variation is not known. Several GAS surface-associated and secreted components were shown to contribute to variation in biofilm [12,13,33]. In addition, transcription regulators, such as Mga, CovR, and Srv are likely to play substantial roles in GAS biofilm formation [11,33] due to their transcriptional regulation of numerous genes. Therefore, it is logical to assume that the combination of genomic/proteomic make up, allelic polymorphisms, and transcription regulation all contribute to this phenomenon. In addition, discrepancies between *in vitro *data obtained with laboratory-stored strains and microcolony formation *in vivo *likely exist and add yet another unknown to the complexity of GAS biofilm/microcolony formation and its role in pathogenesis. Despite this complexity, the analyses involving isogenic strains of the same genetic background provide valuable information that allows assessment of the role and contribution of a given GAS component to biofilm formation.

The M1 MGAS5005 strain was shown to form biofilm *in vitro *and in experimental animals [8,33,53], and the present study demonstrates a significant role of Scl1.1 in this process. Likewise, the MGAS6183 strain, representing M41-type isolates often associated with pyoderma, produced a more robust biofilm biomass under the same experimental conditions and Scl1.41-deficient mutant was found to be an important determinant in this process. Similarly, Scl1.28 protein significantly contributes to a robust biofilm made by the M28-type strain MGAS6143. However, a recent study reported that another surface protein, designated AspA, found in M28-type GAS significantly contributed to biofilm formation [54]. The Δ*aspA *isogenic mutant showed 60% reduction in biofilm formation. The strain MGAS6180, which they used, expresses the same Scl1.28 variant present in the MGAS6143 strain we used; our Scl1.28 mutant showed ~44% reduction in 24 h biofilm. We propose that several surface proteins contribute to biofilm formation by M28-type strains including proteins AspA and Scl1.28, and potentially, proteins F1/SfbI and F2 that are also present in these strains [22]. This redundancy is likely responsible for the observed residual biofilms produced by the AspA- and Scl1.28-deficient mutants.

The observed heterogeneity in biofilm architecture of different GAS strains was previously observed by Lembke *et al. *[28] and was also documented in the current study using FESEM. In addition, here we report the differences in GAS-cell surface morphology and within cell-to-cell junctions in biofilms formed by M1- and M41-type strains. The structural and genetic determination of these differences is not known since M41 genome has not been sequenced, but may be associated with the presence of additional surface proteins, such as the F2 protein [55] encoded by *prtf2 *gene found in this strain [22]. Even more striking was an observed difference in the amount of the extracellular material associated with each strain, referred to as BAEM (bacteria-associated extracellular matrix). It has been shown that extracellular matrix, also called glycocalyx, is produced by biofilm-forming bacteria. DNA, lipids, proteins [33], polysaccharides and dead cell debris [56] were identified in this matrix and for gram-positive bacteria, teichoic acids have also been detected [57,58]. The exopolysaccharide component of the glycocalyx is detected using carbohydrate-binding lectins, such as concanavalin A (ConA) [10]. Both FESEM analysis and ConA staining detected more BAEM associated with M1 biofilm compared to M41, which produced larger biofilm. These observations suggest that GAS biofilm is stabilized differently by different strains and that higher BAEM production does not necessarily pre-determine larger biofilm mass. Consequently, a combination of biofilm features rather than biofilm size alone may be more relevant to pathogenicity of a given GAS strain.

Diminished adherence and biofilm formation could be associated with changes in cell surface hydrophobicity [59] of the *scl1 *mutants. Indeed, the lack of Scl1 resulted in both decreased hydrophobicity and the ability to form biofilm, albeit in a somewhat disproportionate manner. A decrease in the hydrophobicity index by only ~8%, as compared to the wild type-strain, was measured for the M41Δ*scl1 *mutant and this modest decrease was accompanied by a rather large reduction in biofilm formation capacity after 24 h by 30%. Greater decrease in cell-surface hydrophobicity was measured for the M1Δ*scl1 *(~21%) and M28Δ*scl1 *(~22%) mutants, which was accompanied by a significant loss in biofilm formation after 24 h by both isogenic strains by ~55% and ~41% (*P *≤ 0.001 for each comparison), respectively. In addition, heterologous expression of Scl.41 in *L. lactis *increased hydrophobicity index of this organism to ~137% of the WT level, which was accompanied by significant increase in its ability to form biofilm. Similar observations have been reported for the M and M-like protein mutants that typically, but not always, exhibit concurrent loss of both biological features [12]. For example, isogenic ΔMrp49 mutant had a non-significant drop in hydrophobicity (~2%) but significantly lower biofilm formation after 48 h by ~30%, whereas ΔEmm1 mutant lost ~78% hydrophobicity and ~44% biofilm formation capacity. In summary: (i) here we report that the Scl1 adhesin is also a hydrophobin with varying contribution to the overall surface hydrophobicity among GAS strains representing different M types and (ii) Scl1-associated surface hydrophobicity is likely to contribute to Scl1-mediated biofilm formation.

To test whether Scl1 alone could support biofilm formation, we used a heterologous *L. lactis *strain, which provides an expression system for membrane-bound proteins of gram- positive bacteria with LPXTG cell-wall anchoring motifs [39,60-62], including the group A streptococcal M6 protein [38,63]. In a recent study by Maddocks *et al. *[54] it was shown that heterologous expression of AspA GAS surface protein was able to induce a biofilm phenotype in *L. lactis *MG1363. We were also able to achieve a gain-of-function derivative of the *L. lactis *WT MG1363 strain, (MG1363::pSL230), displaying an altered phenotype associated with biofilm formation, as compared to wild-type parental and vector-only controls. These data support our current model that Scl1 protein is an important determinant of GAS biofilm formation.

**Figure 6:**
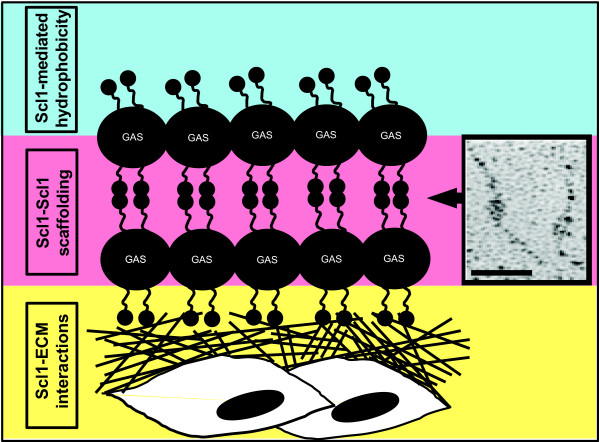
**Scl1-mediated model of GAS biofilm (not to scale)**. Scl1 hydrophobin (current study) initiates bacterial adhesion to animate surfaces [59] within the host (blue field). Scl1 adhesin anchors the growing microcolony by direct binding to tissue extracellular matrix (ECM) components, cellular fibronectin and laminin [19], initiating microcolony formation and anchoring the outside edge of GAS microcolony in tissue (yellow field). Microcolony scaffolding is stabilized by the formation of head-to-head dimers between Scl1 molecules on adjacent chains (pink field). Inset shows Scl1-Scl1 head-to-head dimers formed by rScl1.1 as viewed by electron microscopy after rotary shadowing [64]. *Bar*: 50 nm.

As shown by crystal violet staining and CLSM, biofilm formation by the Scl1-negative mutants was compromised during the initial stage of adherence, as well as microcolony stabilization and maturation. Consequently, their capacity for biofilm formation as compared to the respective WT controls was greatly reduced. This comparison identifies for the first time that the Scl1 protein contributes significantly to biofilm assembly and stability. Based on these observations, as well as previous work by us and others, we propose the following model of Scl1 contribution to GAS tissue microcolony formation (Figure 6). First, the Scl1 hydrophobin (current study) initiates bacterial adhesion to animate surfaces within the host [59]. Next, the Scl1 adhesin anchors the outside edge of growing microcolony in tissue by direct binding to tissue extracellular matrix components, cellular fibronectin and laminin [19]. Microcolony development is stabilized by Scl1-Scl1 scaffolding resulting from Scl1's capacity to form head-to-head dimers [64] between molecules located on adjacent chains. This model will be tested experimentally in future studies.

## Conclusions

In the present work, using pathogenically differing GAS strains, we have demonstrated three concepts. First, we have confirmed previous observations that biofilm formation is an innate property of GAS strains. The M41-type strain used formed a more robust biofilm when compared to M28-type strain as well as M1-type strain. Importantly, the highly invasive M3-type strains devoid of the surface-associated Scl1 also lack the ability to form biofilm. Secondly, the absence of surface-associated Scl1 decreases GAS-cell hydrophobicity suggesting that Scl1 plays a role on the GAS surface as a hydrophobin. Thirdly, we have established that the Scl1 protein is a significant determinant for GAS biofilm formation. This concept was further tested by the heterologous expression of Scl1 in *Lactococcus*, an organism found in dairy fermentation environments, enabling it to form biofilm. Altogether, these data underscore the importance of Scl1 in biofilm-associated regulation of GAS pathogenicity. Recently published work has shown that the recombinant Scl1 binds to the extracellular matrix components, cellular fibronectin and laminin [19]. Our current research provides a foundation warranting additional investigation as to whether direct Scl1-ECM binding may promote GAS biofilm as a bridging mechanism within host tissues.

## Methods

### GAS strains and growth conditions

The wild-type GAS strains M41- MGAS6183, M1- MGAS5005, and M28-type MGAS6143, as well as their *scl1*-inactivated isogenic mutants and complemented M41Δ*scl1 *mutant have been previously described [22,27,65]. In addition, a set of the wild-type M3-type GAS strains MGAS158, MGAS274, MGAS315, MGAS335, MGAS1313, and MGAS2079 was also used. GAS cultures were routinely grown on brain-heart infusion agar (BD Biosciences) and in Todd-Hewitt broth (BD Biosciences) supplemented with 0.2% yeast extract (THY medium) at 37°C in an atmosphere of 5% CO_2_-20% O_2_. Logarithmic phase cultures harvested at the optical density (600 nm) of about 0.5 (OD_600 _~0.5) were used to prepare GAS inocula for biofilm analysis. Colony counts were verified by plating on tryptic soy agar with 5% sheep's blood (Remel). *Lactococcus lactis *subsp. *cremoris *strain MG1363 (provided by Dr. Anton Steen) were grown using M17 broth or agar media (Oxoid) supplemented with 0.5 M sucrose and 0.5% glucose (SGM17 media) at 30°C in an atmosphere of 5% CO_2_-20% O_2_.

### Heterologous Scl1 expression in *Lactococcus lactis*

#### Lactococcus transformation

To obtain electrocompetent cells, 500 ml of SGM17 broth supplemented with 2% glycine was inoculated with an overnight culture and grown until OD_600 _~0.4 was reached. Cells were harvested and washed twice with ice-cold solution A (0.5 M sucrose, 10% glycerol); cells were then re-suspended in solution A (1/1000 of original culture volume) and stored at -80°C [66]. For transformation, cells were thawed on ice and mixed with 1 μl of DNA of the Scl1.41-expressing plasmid pSL230 or pJRS525-vector [22]; and transferred to a cold 1-mm electrode-gap cuvette. Cells were pulsed with 2.0 kV at 25 μF and 400 Ω. Immediately following, suspensions were mixed with 1 ml outgrowth medium (SGM17 broth supplemented with 20 mM MgCl_2 _and 2 mM CaCl_2_) and incubated for 2.5 h before plating on SGM17 agar supplemented with spectinomycin [62].

#### Molecular characterization of transformants

The pSL230 was detected in *Lactococcus lactis *MG1363 transformants by PCR amplification directly from bacterial colonies with *scl1.41*-gene specific primers 232up (5'-CTCCACAAAGAGTGATCAGTC) and 232rev (5'-TTAGTTGTTTTCTTTGCGTTT); pSL230 plasmid DNA was used as a positive control. PCR samples were analyzed on 1% agarose gel in Tris-acetate-EDTA buffer and stained with ethidium bromide. Inocula from colonies *of L. lactis *MG1363, as well as colonies harboring either pJRS525 vector or pSL230 construct were used in subsequent experiments.

#### Western blot analysis

Cell-wall extracts were prepared as previously described [22]. Briefly, cells grown to OD_600 _~0.4 were harvested, washed with TES (10 mM Tris, 1 mM EDTA, 25% Sucrose), re-suspended in TES-LMR (TES containing 1 mg/ml hen egg lysozyme, 0.1 mg/ml mutanolysin, 0.1 mg/ml RNAseA and 1 mM PMSF) and incubated at 37°C for 1 h. After centrifugation at 2500 g for 10 min, the supernatants were precipitated with ice-cold TCA (16% final) at -20°C overnight. Precipitates were rinsed thoroughly with ice-cold acetone and dissolved in 1× sample buffer at 250 μl per unit OD_600_. Samples were subjected to 10% SDS-PAGE, transferred to nitrocellulose, and probed with anti-P176 antiserum followed by goat anti-rabbit-HRP and detected employing chemiluminescent substrate (Pierce).

#### Flow cytometry

Bacterial cells were grown to mid-log phase (OD_600 _~0.4), washed once with filtered DPBS containing 1% FBS and re-suspended in the same buffer. Five million cells were incubated with 1:400 dilution of primary reagents, either rabbit pre-bleed (control) or rabbit anti-P176 antiserum for 30 min on ice, washed with DPBS-FBS and then incubated with 1:200 dilution of second reagent donkey anti-rabbit-APC (Jackson ImmunoResearch) for 30 min on ice. After a final wash and re-suspension in DPBS-FBS, flow cytometric data were acquired with FACSCaliber (BD Biosciences) and analyzed employing FCS Express (De Novo Software).

### Analysis of biofilm formation

#### Crystal violet staining assay

Biofilm formation was tested using tissue culture treated polystyrene 24-well plates. 1.5 ml of logarithmic-phase GAS or *Lactococcus *cultures were seeded without dilution into wells and incubated at 37°C for GAS and 30°C for *Lactococcus *in an atmosphere of 5% CO_2_-20% O_2 _according to indicated time points upon which medium was aspirated. Wells were washed with PBS and 500 μl of 1% crystal violet was added to each well, and incubated at room temperature for 30 min. Dye was then aspirated, wells were washed with PBS, and stain was solubilized with 500 μl of 100% ethanol. Spectrophotometric readings at OD_600 _were recorded for each sample per time point. Samples were analyzed in triplicate in at least three experiments.

#### Confocal laser scanning microscopy (CLSM)

To visualize GAS and *L. lactis *strains by CLSM, bacterial cells were transformed with a GFP-encoding plasmid, pSB027 [67]. 15-mm glass cover slips were placed into 24-well tissue culture plate wells. Logarithmic-phase bacterial cultures were inoculated without dilution and grown for 24 h. Cover slips were rinsed with PBS and fixed with 3% paraformaldehyde at room temperature for 30 min. Biofilms present on cover slips were washed with PBS and mounted onto slides using Prolong Gold mounting media (Invitrogen). Confocal images were acquired using a 63×/1.40 Plan-Apochromat objective and a Zeiss LSM 510 laser scanning confocal on an AxioImager Z1 microscope. An orthogonal view of the Z-stacks was used to display and measure biofilm thickness using Zeiss LSM software. Ten representative images within a single experiment were used to calculate the average vertical thickness measured in micrometers.

To visualize extracellular matrix associated with GAS cells, 24-h biofilm samples were reacted with 100 μg of tetramethyl rhodamine isothiocyanate- (TRITC)-conjugated concanavalin A (TRITC-ConA) (Invitrogen) for 30 min at room temperature in the dark prior to mounting with Prolong Gold medium. An average of ten microscopic views within each sample was reviewed using the 63×/1.40 objective, as described above.

#### Field emission scanning electron microscopy (FESEM)

GAS biofilm samples were grown for 24 h on glass cover slips, washed with PBS, and fixed with 3% paraformaldehyde for 2 h and post-fixed in osmium tetroxide. Samples were next dehydrated in an ethanol gradient, dried using hexamethyldisalizane, mounted onto aluminum stubs and sputter-coated with gold/palladium. The samples were then imaged on a Hitachi S-4800 field emission scanning electron microscope.

### Quantitation of hydrophobicity

A modified hexadecane method [12,37,68] was used to determine the cell hydrophobicity. Briefly, 5 ml of the logarithmic-phase GAS or *Lactococcus *cultures (OD_600 _~0.5) were pelleted, washed and re-suspended in 5 ml of PBS. One ml of hexadecane was added, vortexed for 1 min and incubated for 10 min at 30°C. Mixtures were then vortexed for an additional 1 min and allowed to stand for 2 min for phase separation at room temperature. The absorbance of the lower aqueous phase was read at OD_600 _and compared against the PBS control. Actual hydrophobicity value was calculated using the following equation: Actual Value = [1-(A/A_o_)] × 100, where A is OD_600 _value after hexadecane treatment and A_o _is OD_600 _prior to hexadecane treatment.

### Statistical analysis

Statistical significance was determined using a two-tailed paired Student's *t*-test. The results were considered statistically significant with *P *≤ 0.05 (*) and *P *≤ 0.001 (**).

## Authors' contributions

HO-K is responsible for majority of experiments. ME characterized heterologous expression of Scl1 and BB characterized biofilm formation by M3-type strains. KHM assisted in biofilm analysis using CLSM. DS-B, BJG and HO-K performed FESEM imaging and analysis. SDR provided preliminary results and participated in helpful discussions. SL was the project leader and participated in overall design and coordination of the project. HO-K and SL drafted the manuscript. All authors have read and approved the final manuscript.

## Supplementary Material

Additional file 1 **Figure S1. Biofilm formation by the isogenic wild-type and *scl1*-inactivated GAS strains**. The figure shows gallery views and X-Y orthogonal Z-stack views of GFP-expressing GAS biofilms at 24 h rendered by confocal laser scanning microscopy (CLSM). **Figure S2. Biofilm formation by the wild-type and Scl1-expressing *L. lactis *strains**. The figure shows gallery views and X-Y orthogonal Z-stack views of GFP-expressing *L. lactis *biofilms at 24 h rendered by CLSM. Click here for file
