# Supplementary material for: The streptococcal collagen-like protein-1 (Scl1) is a significant determinant for biofilm formation by group a Streptococcus
Source: BMC Microbiol. 2011 Dec 14;11:262. doi: 10.1186/1471-2180-11-262 (PMC3268755; doi:10.1186/1471-2180-11-262)
Supplement: Additional file 1 — Figure S1. Biofilm formation by the isogenic wild-type and scl1-inactivated GAS strains. The figure shows gallery views and X-Y orthogonal Z-stack views of GFP-expressing GAS biofilms at 24 h rendered by confocal laser scanning microscopy (CLSM). Figure S2. Biofilm formation by the wild-type and Scl1-expressing L. lactis strains. The figure shows gallery views and X-Y orthogonal Z-stack views of GFP-expressing L. lactis biofilms at 24 h rendered by CLSM. [file 1471-2180-11-262-S1.PDF]

Supplementary Material

BMC Microbiology

The streptococcal collagen-like protein-1 (Scl1) is a significant determinant for biofilm formation by group A *Streptococcus*

Heaven A Oliver-Kozup[1], Meenal Elliott[1], Beth A Bachert[‡], Karen H Martin[2, 3],Sean D Reid[4], Diane E Schwegler-Berry[5], Brett J Green[6], Slawomir Lukomski[*1]

Address: [1]Department of Microbiology, Immunology, and Cell Biology, [2]Mary Babb Randolph Cancer Center and [3]Microscope Imaging Facility, West Virginia University School of Medicine, Morgantown, WV, 26506; [4]Department of Microbiology and Immunology, Wake Forest University School of Medicine, Winston-Salem, NC, 27157; and [5]Pathology and Physiology Research Branch and [6]Allergy and Clinical Immunology Branch, Health Effects Laboratory Division, National Institute of Occupational Safety and Health (NIOSH), Morgantown, WV, 26505, USA

[‡]Beth A. Bachert was enrolled in Biomedical Sciences Graduate Programs, West Virginia University Health Sciences Center

*Corresponding author: Slawomir Lukomski

E-mail: slukomski@hsc.wvu.edu

Address: Department of Microbiology, Immunology, and Cell Biology, West Virginia University School of Medicine, 2095 Health Sciences North, PO Box 9177, Morgantown, WV 26506-9177, USA

Figure S1

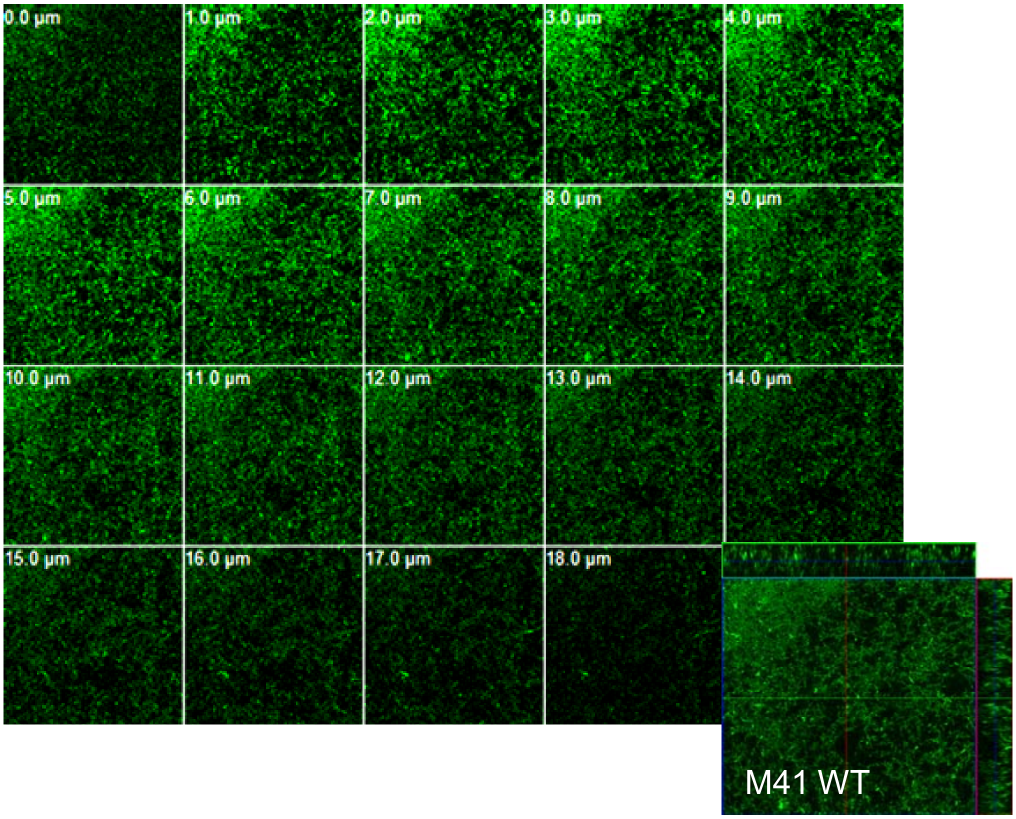

Fig. S1 (a). Confocal laser scanning microscopy (CLSM) of GFP-expressing M41 WT GAS biofilm at 24 h. Panels represent a gallery view of consecutive images taken at 1 micrometer increments. Panel shown in lower right corner represents an X-Y orthogonal Z-stack view (Fig. 4d). Thickness is indicated in micrometers.

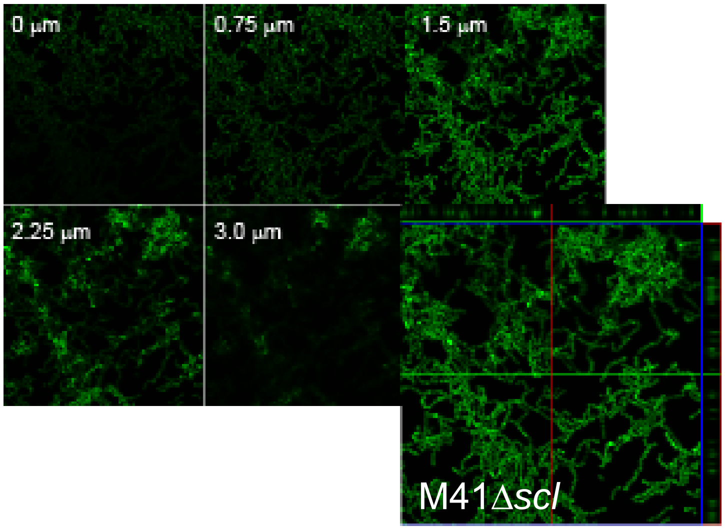

Fig. S1 (b). Confocal laser scanning microscopy (CLSM) of GFP-expressing M41Δ*scl1* GAS biofilm at 24 h. Panels represent a gallery view of consecutive images taken at 0.75 micrometer increments. Panel shown in lower right corner represents an X-Y orthogonal Z-stack view (Fig. 4d). Thickness is indicated in micrometers.

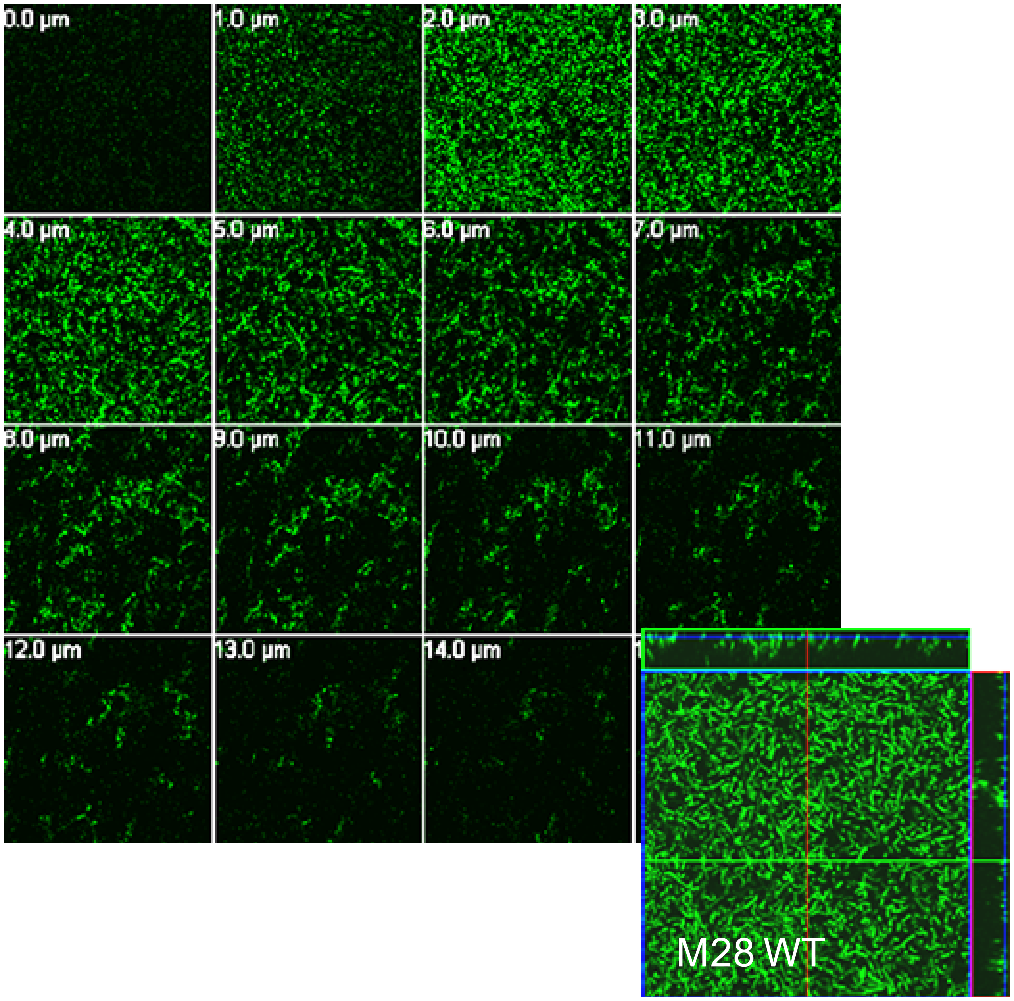

Fig. S1 (c). Confocal laser scanning microscopy (CLSM) of GFP-expressing M28 WT GAS biofilm at 24 h. Panels represent a gallery view of consecutive images taken at 1 micrometer increments. Panel shown in lower right corner represents an X-Y orthogonal Z-stack view (Fig. 4e). Thickness is indicated in micrometers.

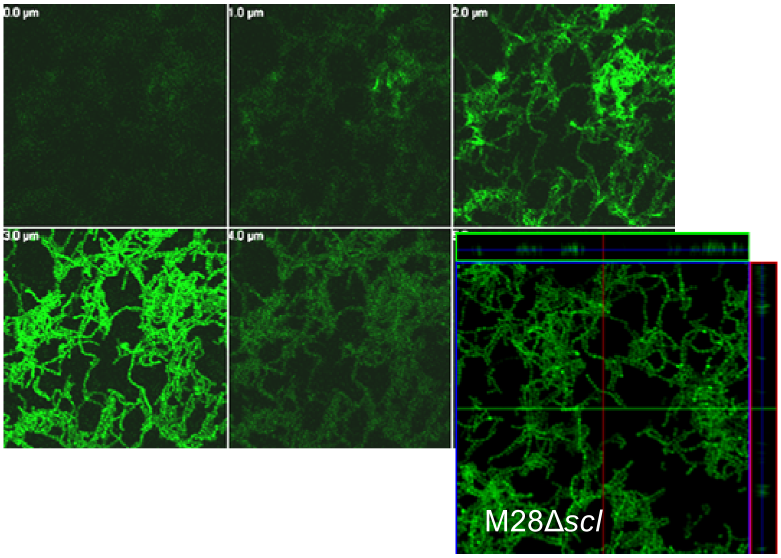

Fig. S1 (d). Confocal laser scanning microscopy (CLSM) of GFP-expressing M28Δ*scl1* GAS biofilm at 24 h. Panels represent a gallery view of consecutive images taken at 1 micrometer increments. Panel shown in lower right corner represents an X-Y orthogonal Z-stack view (Fig. 4e). Thickness is indicated in micrometers.

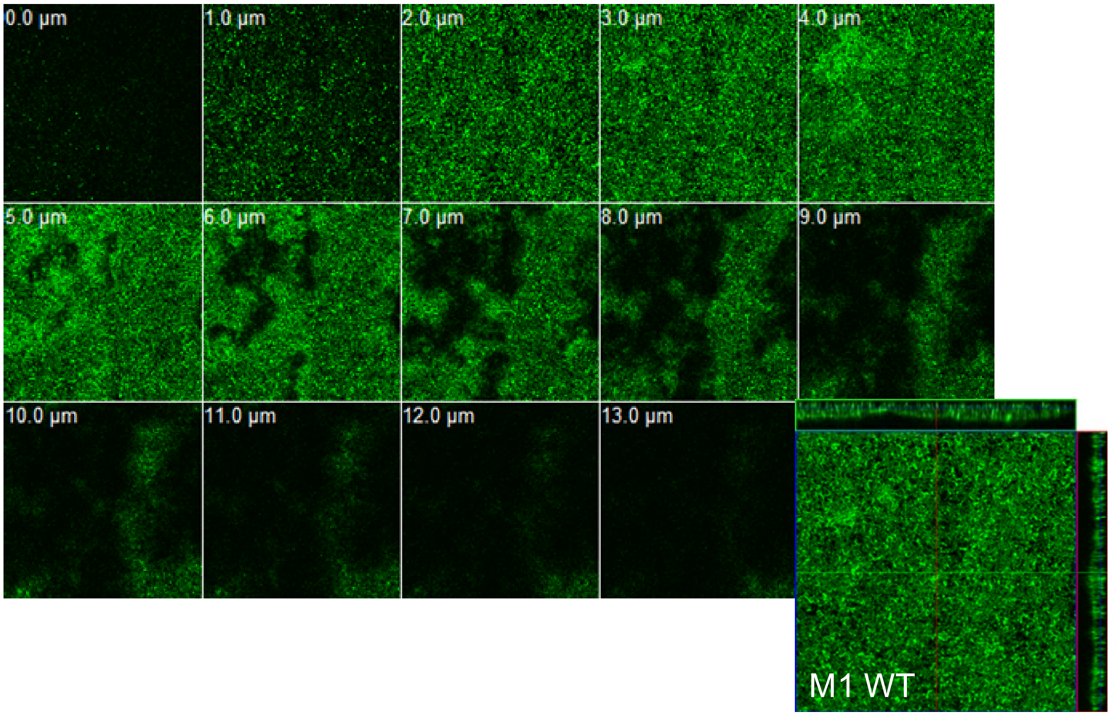

Fig. S1 (e). Confocal laser scanning microscopy (CLSM) of GFP-expressing M1 WT GAS biofilm at 24 h. Panels represent a gallery view of consecutive images taken at 1 micrometer increments. Panel shown in lower right corner represents an X-Y orthogonal Z-stack view (Fig. 4f). Thickness is indicated in micrometers.

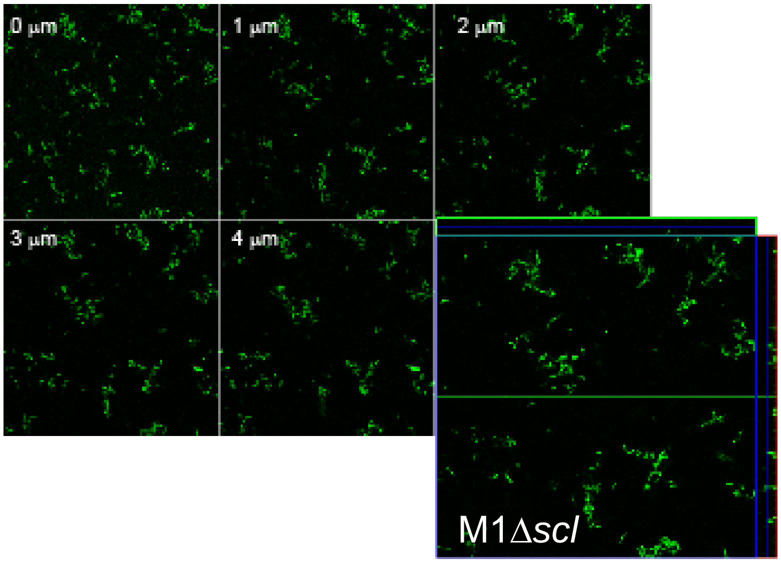

Fig. S1 (d). Confocal laser scanning microscopy (CLSM) of GFP-expressing M1Δ*scl1* GAS biofilm at 24 h. Panels represent a gallery view of consecutive images taken at 1 micrometer increments. Panel shown in lower right corner represents an X-Y orthogonal Z-stack view (Fig. 4f). Thickness is indicated in micrometers.

Figure S2

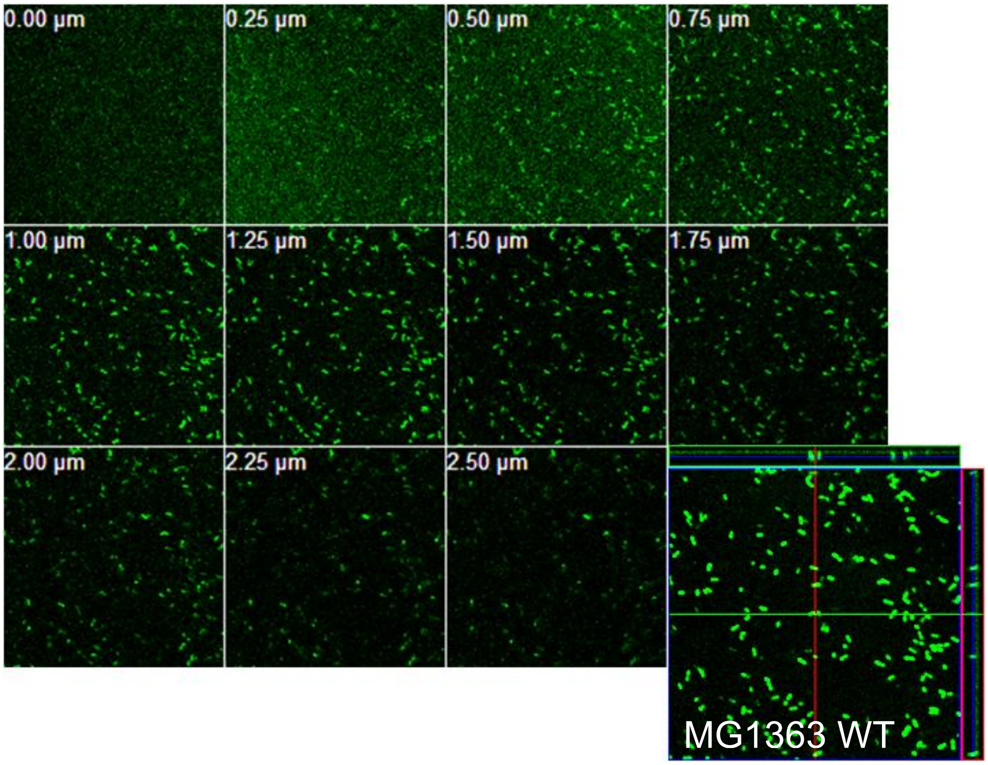

Fig. S2 (a). Confocal laser scanning microscopy (CLSM) of GFP-expressing *Lactococcus lactis* MG1363 WT biofilm at 24 h. Panels represent a gallery view of consecutive images taken at 0.25 micrometer increments. Panel shown in lower right corner represents an X-Y orthogonal Z-stack view (Fig. 5e). Thickness is indicated in micrometers.

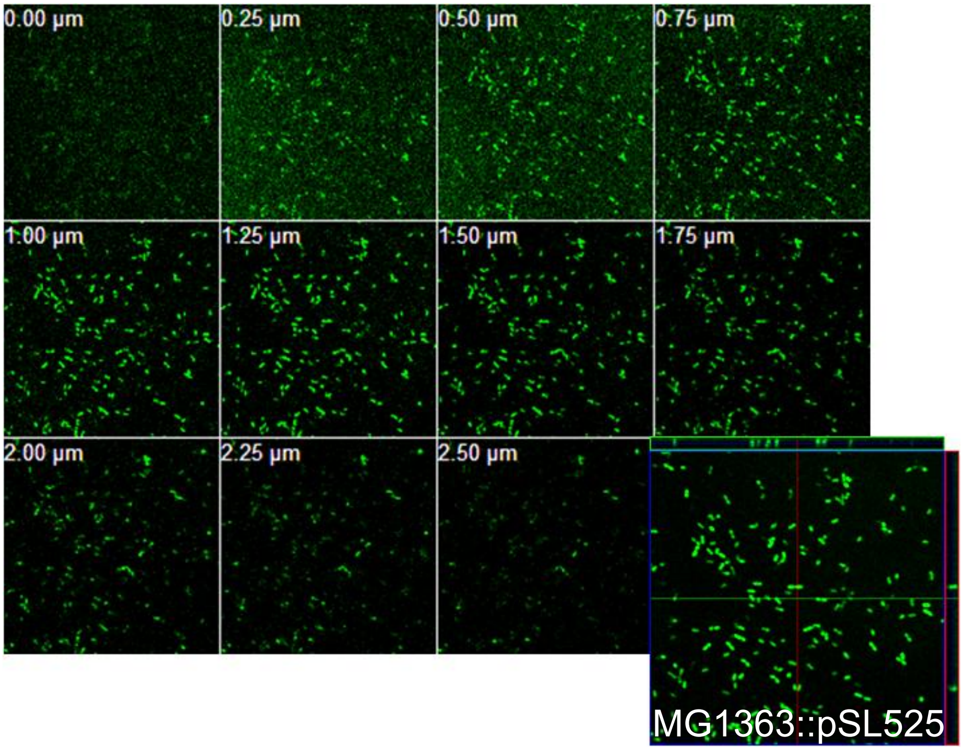

Fig. S2 (b). Confocal laser scanning microscopy (CLSM) of GFP-expressing *Lactococcus lactis* biofilm at 24 h. *L. lactis* was transformed with the shuttle vector pJRS525 (MG1363::pJRS525). Panels represent a gallery view of consecutive images taken at 0.25 micrometer increments. Panel shown in lower right corner represents an X-Y orthogonal Z-stack view (Fig. 5e). Thickness is indicated in micrometers.

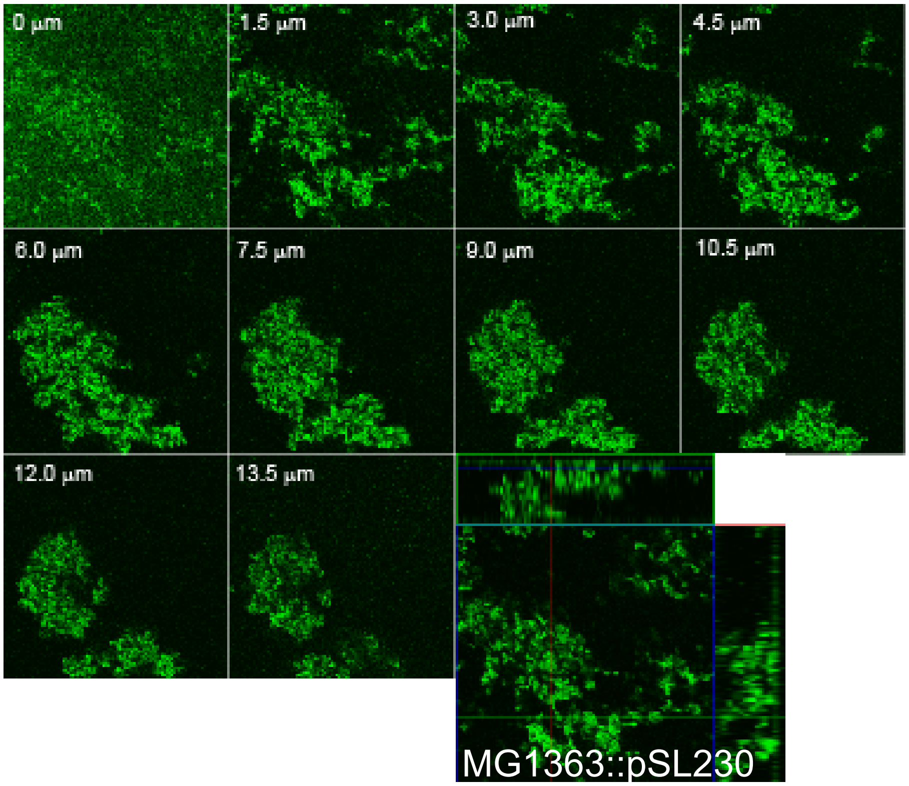

Fig. S2 (c). Confocal laser scanning microscopy (CLSM) of GFP-expressing *Lactococcus lactis* expressing Scl1.41 protein (MG1363::pSL230) biofilm at 24 h. *L. lactis* was transformed with the plasmid construct pSL230 encoding Scl1.41 protein (MG1363::pSL230). Panels represent a gallery view of consecutive images taken at 0.5 micrometer increments. Panel shown in lower right corner represents an X-Y orthogonal Z-stack view (Fig. 5e). Thickness is indicated in micrometers.
